# Tracking the origin of ultralow velocity zones at the base of Earth's mantle

**DOI:** 10.1093/nsr/nwaa308

**Published:** 2021-01-02

**Authors:** Jiuhua Chen

**Affiliations:** Center for the Study of Matters at Extreme Conditions, Department of Mechanical and Materials Engineering, Florida International University, USA

## Abstract

Origins of the ultralow velocity zones may be classified by their velocity-reduction-ratio, R = δ lnVS/δ lnVP, which ranges from 1.2–1.5 for iron oxides or iron-enriched magnesium oxides, 1.6–2.0 for pyrite-type FeO_2_H_x_, 2.3–2.8 for the eutectic melt of Fe + C, 2.7–3.1 for partial melt of (Mg, Fe)SiO_3_ + Fe to 3.5–4.5 for iron-rich post-perovskite.

About three decades ago, seismological models showed that the base of the mantle is laterally heterogeneous, with two vast regions beneath Africa and the Pacific that exhibit lower-than-average seismic velocity, named large low shear velocity provinces (LLSVPs), and a number of scattered patches that exhibit very low seismic velocities of up to 50% lower shear velocity (V_S_) and 25% lower compressional velocity (V_P_) than surrounding materials [1]. These patches, named ultralow velocity zones (ULVZs), are about an order-of-magnitude smaller than the LLSVPs (typically up to 100s and 10s of kilometers in width and height, respectively). Some seismic evidence suggests that the ULVZs may correlate with the locations of hotspots/mantle plumes [2], and therefore the origin of the ULVZs is important for understanding mantle dynamics. To date, the origin of the ULVZs remains an enigma, although partial melting has been accepted as the most possible cause of the ULVZs [3]. Due to the highly differentiated influence of melts on shear and compressional waves, partial melting may cause a high velocity-reduction-ratio between shear and compressional waves (*R* = δ lnV_S_/δ lnV_P_), e.g. 3 : 1 [3]. Seismic observations, on the other hand, indicate that the velocity-reduction-ratio *R* for some ULVZs located outside or at the boundaries of the LLSVPs may be as low as 1 : 1 (Fig. 1). The partial melting hypothesis has difficulty explaining those low-*R* ULVZs. Recent discoveries in high-pressure experiments have revealed significant mineral physics evidence, which may play a critical role for seismologists to track down the origins of different ULVZs.

**Figure 1.**
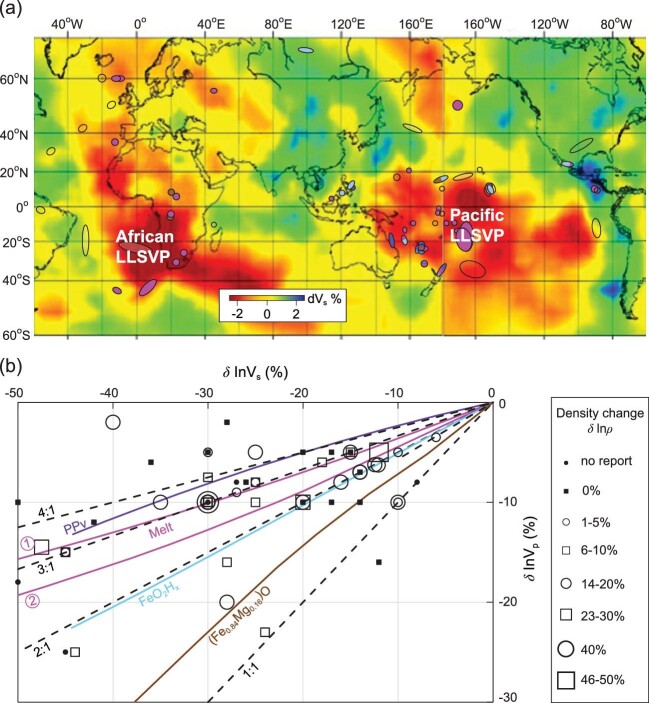
(a) Distribution of global ULVZs and their S to P wave velocity-reduction-ratio (δ lnV_S_/δ lnV_P_). Circles/ovals in purple, pink, light-blue, brown or transparent represent the detected ULVZs with δ lnV_S_/δ lnV_P_ ≈ 4 : 1 (or higher), 3 : 1, 2 : 1, 1 : 1 or undetermined. The background is the tomography based on the shear velocity model GyPSuM [12] at a 2800 km depth showing the Pacific LLSVP, the African LLSVP and the ULVZs with δ lnV_S_/δ lnV_P_ = 2 : 1 (light-blue symbols) at the margins of the LLSVPs. (b) The δ lnV_P_ vs. δ lnV_S_ plot for selected ULSZs shown in (a). The symbol size represents the density change as indicated. Color lines indicate the expected values based on mineral physics data for iron-rich post-perovskite (PPv) [4] in purple, two types of melts (① partial melting of (Mg, Fe)SiO_3 _+ Fe [3] and ② eutectic melting of Fe-C with dihedral angles of 10° [8]) in pink, pyrite-FeO_2_H_x_ (0 < x < 1) [5] in light-blue, and (Fe_0.84_Mg_0.16_)O [7] in brown, mixing with PREM mantle at the core-mantle boundary. Dashed lines represent δ lnV_S_/δ lnV_P_ = 4 : 1, 3 : 1, 2 : 1 and 1 : 1, respectively as indicated.

Besides melt-related causes of the ULVZs [3], several hypotheses of solid phases, such as the iron-rich post-perovskite phase [4], pyrite-type FeO_2_H_x_ (0 < x < 1) [5], iron oxides or iron-enriched magnesium oxides [6,7], have been reported to be able to produce the velocity reductions corresponding to what is observed at the ULVZs. The characteristics of these possible ULVZ origins are summarized in Table >1. Liu *et al.* [8] proposed that the eutectic melting of Fe-C may give rise to some seismic features of an ULVZ. Their data indicate that the eutectic melting with wetting dihedral angles of 10° yields a slightly lower *R* ratio (2.3–2.8) than that of the partial melts (∼3) resulted from (Mg, Fe)SiO_3 _+ Fe [3], whereas non-wetting dihedral angles of 80° may result in a much lower *R* ratio (1.8–1.9). The wetting behavior of Fe-C melt at the lower mantle conditions is not known. If Fe-C melt behaves in a similar way to Fe-S melt, its dihedral angle is likely below 10° at the core-mantle boundary (CMB) [9], and, therefore, *R* > 2.3 is expected for any melt-related ULVZs. For the ULVZs with *R* ratios lower than 2.3, it seems that only certain solid phases may be responsible. Liu *et al.* [5] discovered that pyrite-type FeO_2_H_x_ may reduce seismic S-wave and P-wave velocities down to −42% and −20%, respectively, and increase the density up to 24% with regard to the surrounding ambient mantle. Wicks *et al.* [7] report iron-enriched magnesiowüstite (Fe_0.84_Mg_0.16_)O, reducing V_S_ and V_P_ down to −69% and −57%, respectively, and increasing density up to 40%. The δ lnV_S_ vs. δ lnV_P_ relations calculated based on the published data using the Voigt-Reuss-Hill (VRH) average and Preliminary Reference Earth Model (PREM) are shown in Fig. 1b, indicating that the *R* ratio for pyrite-FeO_2_H_x_ ranges between 1.6 and 2 whereas that for (Fe_0.84_Mg_0.16_)O decreases from about 1.5 down to 1.2 as the volume fraction of (Fe_0.84_Mg_0.16_)O increases. On the other hand, Mao *et al.* [4] demonstrate that iron-rich post-perovskite, (Fe_0.4_Mg_0.6_)SiO_3_, may reduce seismic velocities V_S_ and V_P_ down to −44% and −13%, respectively, yielding an *R* ratio of 3.5 to 4.5 under VRH average (Fig. 1b). The post-perovskite's maximum density increase is estimated to be 7% based on the *in situ* x-ray diffraction data at high pressure/room temperature [4] and the theoretical calculation of thermal expansion [10]. These data from mineral physics, the *R* ratio, in particular, may be critical indicators for establishing the origin of the ULVZs.

**Table 1.** Key features of possible origins of ULVZs.

	Fe-rich oxide (Fe_0.84_Mg_0.16_)O	Pyrite-type FeO_2_H_x_	Melts from C + Fe	Melts from (Mg,Fe)SiO_3 _+ Fe	Post-perovskite (Fe_0.4_Mg_0.6_)SiO_3_
*R*	1.2–1.5	1.6–2.0	2.3–2.8^a^	2.7–3.3	3.5–4.5
Maximum δ lnV_S_, δ lnV_P_	−69%, −57%	−42%, −20%	−100%, −40%^b^	−100%, −40%^b^	−44%, −13%
Maximum δ lnρ	+40%	+24%	+78%	+78%^b^	+7%^c^
References	[7]	[5]	[8]	[3]	[4]

Note: *R* = δ lnV_S_/δ lnV_P_; V_S_: shear-wave velocity; V_P_: compressional-wave velocity; ρ: density. ^a^For dihedral angles of 10°. ^b^Calculated assuming 100% outer core melt. ^c^Combined with the thermal expansion from Ref. [10].

As shown in Fig. 1, most of the observed ULVZs are characterized by *R* ≈ 3, indicating that most of the ULVZs likely originate from partial melting. At the margins of the Pacific LLSVP and/or near the old subduction slabs, a few ULVZs are reported to have a low *R* ratio of 2 or even 1. Primordial thermochemical models of mantle convection [1] show that temperatures inside the LLSVPs are higher than that of the surrounding mantle. The lower temperature at the LLSVP boundaries is unfavorable for melting. Based on their high-pressure experimental results, Mao *et al.* [11] propose that when a subduction slab reaches the CMB, the water carried down by the slab reacts with the nearly inexhaustible iron in the core, producing patches of FeO_2_H_x_ at the base of the lower mantle. These advances of mineral physics knowledge led to the reasonable speculation that the solid phase pyrite-FeO_2_H_x_ is the origin of those ULVZs with *R* = 2. On the other hand, the solid phase of iron-rich post-perovskite may explain some of the ULVZs with *R* > 3 located at lower temperature zones outside the LLSVPs, as shown in Fig. 1.

Noticeably, some ULVZs do not locate on the δ lnV_P_ vs. δ lnV_S_ curves of the proposed origins, i.e. those with *R* > 4.5 or *R *= 1 (Fig. 1b). While simultaneously varying the iron partitioning and phase fraction among the lower mantle constituents (bridgmanite, magnesiowüstite and calcium silicate perovskite) may further tune the *R* ratio to match the values reported for those ULVZs [13], there are trade-offs among the absolute velocity levels, density change and thickness of the ULVZs in seismic models [14]. During seismic interpretation and inversion, the *R* ratio is often preset to a commonly considered value. Some previous studies selectively chose *R* = 3 and 1 for seismic inversion because these *R* ratios had been believed to be the most reasonable choices of possible ULVZ origins. In reality, two similar *R* values, e.g. 1 and 1.2 or even 2, may produce equally good fitting between the seismic model and observation within the current seismogram resolution due to their trade-offs with the density change and the thickness of the ULVZ. Therefore, these experimental mineral physics advances offer essential guidance (Table 1) to seismic modeling. The different characters of the possible ULVZ origins provided by mineral physics studies will help seismologists track the origins of ULVZs and unveil the enigma of seismic heterogeneity at the base of the lower mantle.
